# E-selectin as a prognostic factor of patients hospitalized due to acute inflammatory respiratory diseases: a single institutional study

**DOI:** 10.17179/excli2019-1624

**Published:** 2019-11-11

**Authors:** Hiroshi Nakano, Sumito Inoue, Yoko Shibata, Koya Abe, Hiroaki Murano, Sujeong Yang, Hiroyoshi Machida, Kento Sato, Chisa Sato, Takako Nemoto, Michiko Nishiwaki, Tomomi Kimura, Keiko Yamauchi, Masamichi Sato, Akira Igarashi, Yoshikane Tokairin, Masafumi Watanabe

**Affiliations:** 1 Department of Cardiology, Pulmonology, and Nephrology, Yamagata University Faculty of Medicine, Yamagata, Japan; 2 Department of Pulmonary Medicine, Fukushima Medical University, Fukushima, Japan

**Keywords:** acute inflammatory respiratory disease, infectious pneumonia, interstitial pneumonia, endothelial selectin, prognosis

## Abstract

When examining patients with acute inflammatory respiratory diseases, it is difficult to distinguish between infectious pneumonia and interstitial pneumonia and predict patient prognosis at the beginning of treatment. In this study, we assessed whether endothelial selectin (E-selectin) predicts the outcome of patients with acute inflammatory respiratory diseases. We measured E-selectin serum levels in 101 patients who were admitted to our respiratory care unit between January 2013 and December 2013 because of acute inflammatory respiratory diseases that were eventually diagnosed as interstitial pneumonia (n = 38) and lower respiratory tract infection (n = 63). Seven of these patients (n = 101) died. The pneumonia severity score was significantly higher and the oxygen saturation of arterial blood measured by pulse oximeter (SpO_2_)/fraction of inspiratory oxygen (FiO_2_) was significantly lower in the deceased patients than in the surviving patients. There were significantly fewer peripheral lymphocytes and significantly higher E-selectin serum levels in the deceased patients than in the surviving patients. In the multiple logistic regression analysis, the E-selectin serum levels and SpO_2_/FiO_2_ ratio were independent predictive factors of prognosis. The risk of death during acute respiratory disease was determined using a receiver operating characteristic (ROC) curve analysis. The area under the curve (AUC) was 0.871 as calculated from the ES, and the cutoff value was 6453.04 pg/ml, with a sensitivity of 1.00 and a specificity of 0.72 (p = 0.0027). E-selectin may be a useful biomarker for predicting the prognosis of patients with acute inflammatory respiratory diseases.

## Introduction

Despite progress in medical treatments such as antibiotic therapy and the development of public health, respiratory diseases are one of the leading causes of death worldwide (Lozano et al., 2013[10]). Pneumonia is a major respiratory disease and a life-threatening health problem. Moreover, in Japan, more than 100,000 people die from pneumonia every year, making it the third leading cause of death (Ishiguro et al., 2016[8]). Interstitial pneumonia is also a lethal disease. The prognosis of interstitial pneumonia is extremely poor. The mortality ratio of acute exacerbation of idiopathic pulmonary fibrosis (IPF), the most significant disease among interstitial pneumonia, is 80 % (Enomoto et al., 2018[6]; Atsumi et al., 2018[1]; Marchioni et al., 2018[11]). Moreover, 40 % of patients with IPF die from acute exacerbation (Natsuizaka et al., 2014[13]).

We can evaluate severity and predict the prognosis of community-acquired pneumonia (CAP) using A-DROP scores established by the Japanese Thoracic Society. Patients are scored by age, presence of dehydration, presence of respiratory failure, state of consciousness or orientation, and blood pressure (Shindo et al., 2008[18]). However, the A-DROP score is not used for evaluating the severity of acute exacerbation of interstitial pneumonia. Instead, Krebs von den Lungen-6 (KL-6), Surfactant protein (SP)-A, and SP-D are effective biomarkers for evaluating the severity and prognosis of interstitial pneumonia (Yokoyama et al., 2006[20]; Chiba et al., 2018[4]). Clinically, we examine many patients with acute respiratory failure such as pneumonia and interstitial pneumonia. It can be difficult to distinguish between bacterial pneumonia and interstitial pneumonia and to predict prognosis at the time of admission. Therefore, in situations where pneumonia and interstitial pneumonia cannot be distinguished, useful biomarkers are necessary to estimate severity and prognosis.

Endothelial selectin (E-selectin) is an adhesion molecule expressed on vascular endothelial cells. Pro-inflammatory cytokines such as tumor necrosis factor (TNF)-α or interleukin-1β induce E-selectin (Darveau et al., 1995[5]). E-selectin is a mediator of leukocyte rolling and recruitment during infection. E-selectin plays an important role in the attachment of leukocytes to endothelial cells and the accumulation of leukocytes in inflamed tissues during infectious diseases, malignant tumors, and autoimmune diseases (Darveau et al., 1995[5]; Benekli et al., 1998[2]; McMurray, 1996[12]). We demonstrated previously that E-selectin predicts the occurrence of acute lung injury (ALI) in pneumonic patients. E-selectin serum levels were elevated in pneumonic patients with ALI or clinically comparable ALI (cALI) (Osaka et al., 2011[16]). We investigated whether evaluating the circulating serum E-selectin levels in patients with severe pneumonia can be useful for the prediction of complicating ALI or cALI (Osaka et al., 2011[16]). Based on this foundation, we hypothesized that E-selectin plays an important role in the severity of acute inflammatory respiratory diseases such as infectious pneumonia and interstitial pneumonia. In this study, we examined whether E-selectin is a biomarker for estimating the severity and prognosis before differential diagnosis in the acute phase of acute inflammatory respiratory diseases such as pneumonia and interstitial pneumonia.

## Material and Methods

### Study population

We recruited 101 patients admitted to Yamagata University Hospital between January 2013 and December 2013 because of acute inflammatory respiratory diseases (63 patients were finally diagnosed with bacterial pneumonia and 38 patients were finally diagnosed with interstitial pneumonia). Patients with chronic obstructive pulmonary disease (COPD) exacerbation were excluded because non-inflammatory etiologies such as heart failure were sometimes associated with exacerbation, even in patients with COPD exacerbation, because of lower respiratory infection. The Institutional Ethics Committee of the Yamagata University Faculty of Medicine approved this study (approval number; H23-134, approval date Jan. 5^th^, 2012; H28-221, approval date Aug. 29^th^, 2016). Written informed consent was obtained from all subjects. The severity of CAP was evaluated in all patients using the “A-DROP” score (patient age, presence of dehydration, presence of respiratory failure, state of consciousness/orientation, and presence of shock (low blood pressure)), established by the Japanese Respiratory Society (Shindo et al., 2008[18]). At the time of admission, all patients underwent chest Computed tomography (CT) scans for the diagnosis of acute inflammatory respiratory diseases. Sputum and/or blood cultures were performed for the patients from whom samples could be obtained. The final diagnosis of bacterial pneumonia or interstitial pneumonia was performed by multiple respiratory physicians with reference to the CT image findings, laboratory data including sputum and blood bacterial cultures, and clinical course. Although all patients received suitable treatment according to the final diagnosis, seven patients died.

### Laboratory data

Peripheral blood counts, nutritional status (as indicated by serum total protein (TP) levels), albumin levels, liver function, renal function, inflammatory response (indicated by C-reactive protein (CRP) levels), procalcitonin (PCT) as a biomarker of bacterial infection or sepsis, and KL-6 as a biomarker of interstitial pneumonia were measured at the time of admission using routine laboratory tests. Serum samples were collected simultaneously with the blood collection at the time of admission and stored in a freezer at −80 °C.

### Measurement of E-selectin serum levels

E-selectin serum concentrations were measured with enzyme-linked immunosorbent assay kits (R&D Systems, Minneapolis, MN), according to the manufacturer's protocol.

### Statistical analyses

All data were expressed as the mean ± standard deviation or the median and 25^th^ to 75^th^ percentiles. To compare between two groups, the Mann-Whitney U test or the Chi-squared test were conducted. Risk factors for death were detected by univariate and multivariate logistic regression analyses. We used a receiver operating characteristic (ROC) curve to determine the cutoff value for E-selectin serum levels in detecting the risk of death in patients with acute inflammatory respiratory diseases. All statistical analyses were performed with JMP software, version 11.0.0 (SAS Institute Inc., Cary, NC, USA), and p < 0.05 was considered statistically significant.

## Results

Table 1(Tab. 1) summarizes comparisons of the patient profiles, illness severity according to A-DROP score, oxygenation, and laboratory data between the surviving patients and deceased patients. There were no significant differences in age, sex, peripheral white blood cell numbers, nutrition status, liver function, or renal function between the surviving patients and deceased patients. More of the patients who died had required oxygen supplementation. Lower SpO_2_/FiO_2_ levels and higher A-DROP scores were reported in the deceased patients compared to the surviving patients. Furthermore, there were significantly fewer peripheral lymphocytes in the deceased patients than in the surviving patients. Although the inflammatory responses such as CRP, PCT, and KL-6 serum levels did not differ between the two groups, the E-selectin serum levels were significantly higher in the deceased patients than in the surviving patients.

The univariate logistic regression analysis was used to evaluate the risk of death in patients with acute inflammatory respiratory diseases. Fewer peripheral blood lymphocytes, higher E-selectin serum levels, lower SpO_2_/FiO_2 _ratios, and higher A-DROP scores were significant predictive factors for death (Table 2(Tab. 2)).

In the multiple logistic regression analysis, a lower SpO_2_/FiO_2_ ratio and a higher E-selectin serum level were significant risk factors for predicting death even when adjusted for age, sex, peripheral lymphocyte number, and illness severity (Table 3(Tab. 3)).

**Figure 1:**
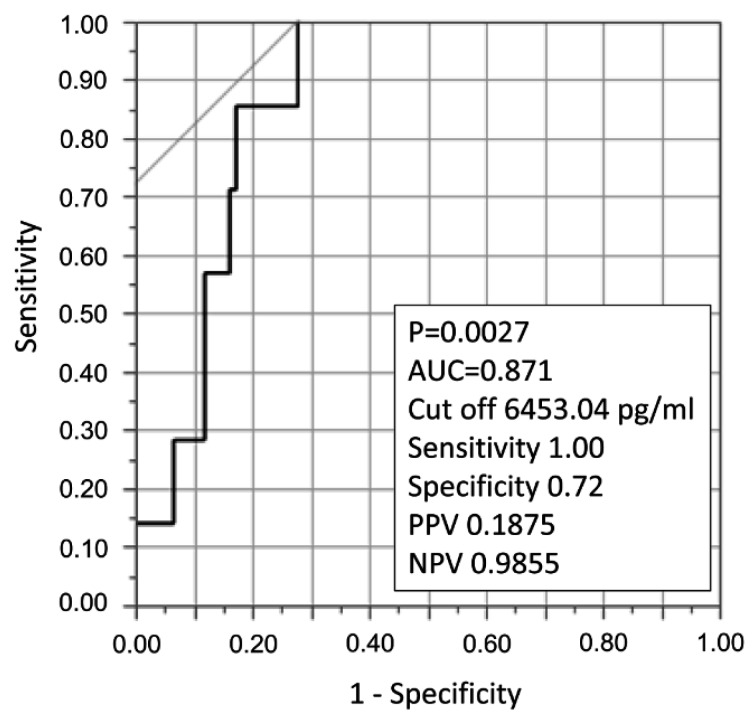
ROC curve to detect an E-selectin cutoff value for predicting death in patients with acute inflammatory respiratory diseases. The area under the curve (AUC) was 0.87, and the cutoff value was 6453.04 pg/ml, with a sensitivity of 1.00 and a specificity of 0.72 (p = 0.0027). Positive predictive value (PPV) was 0.1875, and negative predictive value (NPV) was 0.9855.

We plotted a ROC curve to detect the E-selectin cutoff value for predicting death in patients with acute inflammatory respiratory diseases (Figure 1(Fig. 1)). The area under the curve (AUC) was 0.87, and the cutoff value was 6453.04 pg/ml, with a sensitivity of 1.00 and a specificity of 0.72 (p = 0.0027). Positive predictive value was 0.1875, and negative predictive value was 0.9855.

## Discussion

In this study, we investigated whether E-selectin serum levels can serve as an independent predictive marker for the prognosis of patients with acute inflammatory respiratory diseases, including infectious pneumonia and interstitial pneumonia. Various factors predicting prognosis for infectious pneumonia or interstitial pneumonia have been investigated in previous studies, and several biomarkers have been established as predictors of prognosis for each disease (Shindo et al., 2008[18]; Chalmers et al., 2010[3]; Zhang et al., 2018[21]; Wang et al., 2017[19]; Gwak et al., 2015[7]). For example, the A-DROP score is a well-known evaluation system to predict the severity and prognosis of CAP in Japan (Shindo et al., 2008[18]). In addition to the A-DROP score, CURB65 and the pneumonia severity index (PSI) are also similar evaluation systems (Chalmers et al., 2010[3]; Zhang et al., 2018[21]). Recently, a combination of data including renal function, white blood cell count, CRP levels, and serum HCO_3_^-^ was an independent predictive factor for hospital mortality (Wang et al., 2017[19]). The serum lactate level was reported to be associated with inpatient mortality in patients with CAP (Gwak et al., 2015[7]). KL-6 is a good predictive marker for the prognosis of interstitial pneumonia (Yokoyama et al., 2006[20]). The serum ferritin level has a good prognostic value in patients with acute exacerbation of IPF, which is the most common type of idiopathic interstitial pneumonia (Enomoto et al., 2018[6]). The gender-age-physiology (GAP) index, composed of sex, age, and respiratory function, is known to predict the prognosis of interstitial pneumonia (Atsumi et al., 2018[1]). Based on these findings, many factors have been reported to predict the prognosis of each disease, but few factors are common predictors of both diseases. Therefore, pulmonary physicians must correctly differentiate and diagnose infectious pneumonia and interstitial pneumonia to utilize appropriate biomarkers and select the best treatment for these diseases. In the initial examination of patients with respiratory diseases, thoracic radiographic imaging such as chest X-ray and chest CT scan and blood sampling tests are usually performed. We can make a differential diagnosis if typical radiographic imaging or laboratory data findings are observed. However, clinically, it is sometimes difficult to distinguish between infectious pneumonia and interstitial pneumonia if typical findings are not present. Pulmonary physicians must make a diagnosis and initiate treatment for patients with acute inflammatory respiratory diseases even when lacking sufficient information for a correct diagnosis. Based on these findings, identifying common biomarkers between infectious pneumonia and interstitial pneumonia that can estimate prognosis is necessary.

In this study, a lower SpO_2_/FiO_2_ ratio was also an independent predictive marker for prognosis in patients with acute inflammatory respiratory diseases. The presence of respiratory failure is the one of the evaluation values in the A-DROP score and CURB-65 score. However, SpO_2_ tends to fluctuate depending on measurement conditions. Therefore, E-selectin may be a more accurate biomarker to predict prognosis in patients with acute inflammatory respiratory diseases than the SpO_2_ value even though the measured SpO_2_ value can be determined immediately.

Osaka et al. investigated whether circulating levels of E-selectin were elevated in pneumonic patients with ALI or cALI. The measurement of E-selectin serum levels in patients with severe pneumonia may be useful for discriminating between complicating ALI or cALI (Osaka et al., 2011[16]). This study showed that E-selectin is a more effective biomarker for predicting the onset of ALI than white blood cell count, CRP, and LDH, which have been well-known biomarkers for a long time. Moreover, circulating levels of soluble E-selectin are elevated in patients with sepsis and shock (Kayal et al., 1998[9]; Newman et al., 1993[14]; Reinhart et al., 2002[17]). Additionally, hypoxia is more common in patients with high soluble E-selectin levels and systemic inflammatory response syndrome (SIRS) compared to patients with normal E-selectin levels and SIRS (Okajima et al., 2006[15]). In our study, we showed that the serum E-selectin level is an independent predictor of prognosis in patients with acute respiratory disease, including infectious pneumonia and interstitial pneumonia. Even in cases in which pulmonary physicians cannot determine whether the patient has bacterial or interstitial pneumonia at the time of admission, it is worthwhile to predict their prognosis using a single factor, E-selectin.

This study has several limitations. We retrospectively performed this study at a single institute, and the study size was fairly small. All patients with interstitial pneumonia were clinically diagnosed by laboratory data, such as the KL-6 level, and radiological findings, such as chest X-ray and chest CT scan; pathological findings were not used for diagnosis. E-selectin was only measured once during days one to four. The time courses of E-selectin levels were not evaluated. Information regarding the long-term prognosis throughout the hospitalization period was not available. Further investigation will be necessary to solidify the clinical significance of E-selectin in acute respiratory disease by evaluating the time course of E-selectin.

## Conclusions

In conclusion, we demonstrated that E-selectin is an independent predictive factor for death from acute respiratory diseases including infectious and interstitial pneumonia. These results suggest that E-selectin might be a useful biomarker for predicting the prognosis of patients with acute respiratory diseases, even in situations in which pulmonary physicians cannot determine whether the patient has bacterial or interstitial pneumonia.

## Acknowledgements

The authors would like to thank Enago (www.enago.jp) for the English language review.

## Competing interest

HN received lecture fees from Pfizer Japan Inc., Boehringer Ingelheim Japan, AstraZeneca K.K. SI received lecture fees from Otsuka Pharmaceutical Co., Ltd, Novartis Pharma K.K., AstraZeneca K.K., GlaxoSmithKline K.K., Chugai Pharmaceutical Co., Ltd., Boehringer Ingelheim Japan, MSD K.K., ONO PHARMACEUTICAL CO., LTD., Meiji Seika Pharma Co., Ltd., KYORIN Pharmaceutical Co., Ltd., Taiho Pharmaceutical Co., Ltd., Eli Lilly Japan K.K., NIHON PHARMACEUTICAL CO., LTD., TEIJIN PHARMA LIMITED., Pfizer Japan Inc., DAIICHI SANKYO COMPANY, LIMITED, and received research grant from Novartis Pharma K.K., Chugai Pharmaceutical Co., Ltd., MSD K.K., KYORIN Pharmaceutical Co., Ltd., and JSPS KAKENHI (Grant Number JP 26461153). YS received lecture fees from Boehringer Ingelheim Japan, AstraZeneca K.K., Novartis Pharma K.K., Taisho Toyama Pharmaceutical Co., Ltd., Meiji Seika Pharma Co., Ltd., TEIJIN PHARMA LIMITED., MSD K.K., ONO PHARMACEUTICAL CO., LTD., KYORIN Pharmaceutical Co., Ltd., Chugai Pharmaceutical Co., Ltd., Eli Lilly Japan K.K., Pfizer Japan Inc., and received research grant from MSD K.K., Boehringer Ingelheim Japan, TEIJIN PHARMA LIMITED, Novartis Pharma K.K., JSPS KAKENHI (Grant Number JP 26461177). SY received lecture fees from Boehringer Ingelheim Japan, AstraZeneca K.K. HM received lecture fees from ONO PHARMACEUTICAL CO., LTD., Boehringer Ingelheim Japan. KS received lecture fee from CHUGAI PHARMACEUTICAL CO., LTD. TN received lecture fee from Teijin Pharma Ltd Japan. TK received lecture fee from ONO PHARMACEUTICAL CO., LTD., and received research grant from JSPS KAKENHI (Grant Number JP 15K19411). KY received lecture fee from Pfizer Japan Inc. MS received lecture fees from AstraZeneca K.K., Boehringer Ingelheim Japan. YT received lecture fees from MSD K.K., Pfizer Japan Inc., Astellas Pharma Inc., Taisho Toyama Pharmaceutical Co., Ltd., Chugai Pharmaceutical Co., Ltd. MW received lecture fees from Bayer Yakuhin, Ltd., Otsuka Pharmaceutical Co., Ltd., Nippon Boehringer Ingelheim Co., Ltd., DAIICHI SANKYO COMPANY, LIMITED, and received research grant from MEDTRONIC JAPAN CO., LTD., Astellas Pharma Inc., ONO PHARMACEUTICAL CO., LTD., CHUGAI PHARMACEUTICAL CO., LTD., Bayer Yakuhin, Ltd.

## Sources of funding

This research was not funded from anywhere.

## Contributors

Conception: YS. Study design: HM, SI, YS, TK, Making data base: TK, Data collection: KS, KA, HM, SY, HN, MS, TN, CS, MN, KY, AI, YT, Data analysis: HM, SI, YS, TK, KS, MW, Data interpretation: all authors. Writing and reviewing the manuscript: all authors. Final approval of the manuscript: all authors.

## Figures and Tables

**Table 1:**
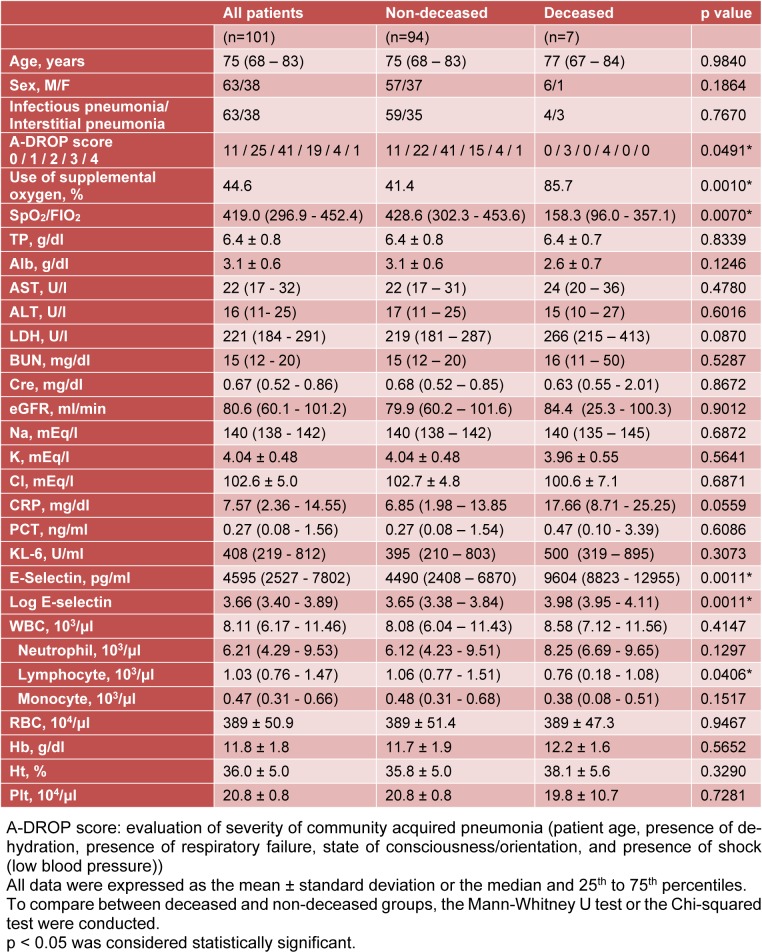
Profiles of patients and data, comparison of deceased and non-deceased patients

**Table 2:**
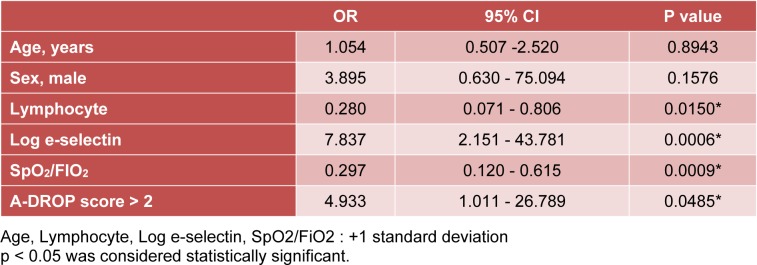
Univariate logistic regression analysis investigating potential risk factors associated with poor prognosis in patients with acute inflammatory respiratory diseases

**Table 3:**
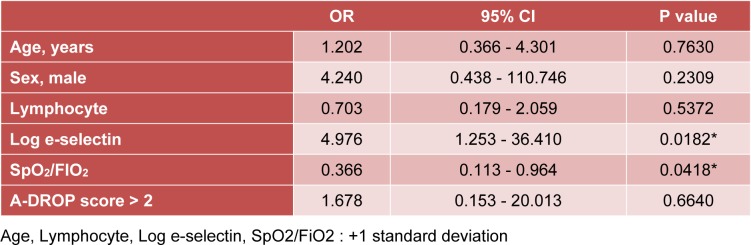
Multivariate logistic regression analysis investigating potential risk factors associated with poor prognosis in patients with acute inflammatory respiratory diseases
